# Would you feel comfortable leaving your relative alone for 2 days without any help from others?

**DOI:** 10.1002/alz70858_097004

**Published:** 2025-12-24

**Authors:** Huong Q Nguyen, Annette Langer‐Gould, Eric A Lee, Soo Borson, Michelle Jurado, Beatriz M Alviso, Mayra Macias, Katherine L. Possin

**Affiliations:** ^1^ Kaiser Permanente Southern California, Pasadena, CA, USA; ^2^ Kaiser Permanente Southern California, Los Angeles, CA, USA; ^3^ University of Washington, Seattle, WA, USA; ^4^ Memory and Aging Center, Weill Institute for Neurosciences, University of California, San Francisco, San Francisco, CA, USA; ^5^ Global Brain Health Institute (GBHI), University of California San Francisco (UCSF); & Trinity College Dublin, San Francisco, CA, USA

## Abstract

**Background:**

While population norms for cognitive tests are well established, none exists for assessing daily function that could easily and quickly assist busy physicians in determining if patients have mild (mild cognitive impairment, MCI) or major (dementia) neurocognitive disorder. The purpose of this analysis is to describe test characteristics of a simple question directed at patients’ family and friend care partners to assist primary care in distinguishing between MCI and dementia.

**Methods:**

Patients 65 and older with memory concerns were referred by their primary care physicians to the Brain Health Assessment (BHA) care pathway as part of a large pragmatic trial to test a practice bundle to improve earlier detection of cognitive impairment in primary care within an integrated healthcare system serving diverse patients (53% completed the assessment in Spanish). Registered nurses assisted physicians with the work‐up for memory concerns by synthesizing information gathered from objective cognitive testing with the TabCAT‐BHA, assessments of patients’ function via their nominated care partners (instrumental and basic activities of daily living), and chart review to arrive at a preliminary classification of whether patients had MCI or dementia. Care partners were asked whether they “would feel comfortable leaving (their) relative alone for 2 days without any help from others?”. We calculated standard test characteristics for this question with the nurses’ preliminary staging.

**Results:**

440 care partners completed the functional assessment interview and provided a response to the question. Of 155 patients with a preliminary staging of dementia, 130 (84%) care partners endorsed not feeling comfortable leaving their relative alone compared to 36 (13%) of 285 care partners of patients with MCI. For patients with dementia, spouses were only slightly more likely (89%) to provide positive endorsement the question compared to adult children (85%). Overall, this question has high sensitivity (84%), specificity (87%), positive (78%) and negative (91%) predictive value for distinguishing dementia from MCI.

**Conclusions:**

Although care partners’ social desirability is a consideration, a simple question could have high utility for busy primary care providers to not only feel confident to diagnose dementia but also, help facilitate care and safety planning for the patient.

